# Preoperative Femoral Nerve Block and Postoperative Sciatic Nerve Block at the Subgluteal Space After Total Knee Arthroplasty: A Retrospective Cohort Study

**DOI:** 10.7759/cureus.50882

**Published:** 2023-12-21

**Authors:** Yuki Okutomi, Yasutaka Konishi, Akihito Kakinuma, Shigehito Sawamura

**Affiliations:** 1 Department of Anesthesia and Critical Care, Teikyo University School of Medicine, Tokyo, JPN

**Keywords:** vomiting, total knee arthroplasty, subgluteal space, sciatic nerve block, nausea

## Abstract

Background

A preoperative sciatic nerve block (SNB) before total knee arthroplasty (TKA) frequently causes postoperative drop foot; however, this can also occur as an unintended result of surgical invasion. This study assessed the benefits of a postoperative SNB at the subgluteal space for patients who underwent TKA.

Methodology

This was a single-center, retrospective cohort study. Patients who underwent TKA under general anesthesia between May 2018 and June 2019 at the Teikyo University School of Medicine were screened for inclusion. They received either a preoperative femoral nerve block alone (control group; n = 87) or a preoperative femoral nerve block and postoperative SNB at the subgluteal space (post-SNB group; n = 40). The primary outcome was the pain-related Numerical Rating Scale (NRS) scores. The secondary outcomes were postoperative nausea and vomiting (PONV), intravenous patient-controlled analgesia (iv-PCA) suspension, and postoperative complications.

Results

No significant differences were observed in the characteristics, NRS scores, time to first drug use for pain, and iv-PCA suspension between groups. However, the incidence of PONV was significantly lower in the post-SNB group (p = 0.03). Logistic regression analysis revealed that droperidol doses of iv-PCA and the presence of postoperative SNB were independently associated with PONV occurrence [A1] {(p = 0.008, 95% confidence intervals (CI) [0.46, 0.89] and (p = 0.02, 95% CI [0.25, 0.88])}.

Conclusions

A postoperative SNB at the subgluteal space following TKA does not improve postoperative pain control; however, it may have contributed to reduced PONV.

## Introduction

Postoperative pain associated with total knee arthroplasty (TKA) is mainly categorized as pain of the anterior knee or posterior knee innervated by the femoral and sciatic nerve, respectively [1]. Inadequate analgesia can lead to postoperative pain, inhibit rehabilitation, and decrease the quality of life [2-5]. Therefore, narcotic analgesia is generally used as a multimodal analgesic approach to improve postoperative pain [5]. However, narcotic analgesia often results in postoperative nausea and vomiting (PONV), respiratory depression, constipation, etc., which not only causes considerable discomfort and emotional distress to patients but also increases the risks of immobilization and delayed hospital discharge [5,6].

The use of a peripheral nerve block is a part of multimodal analgesia [6,7]. However, even though a femoral nerve block (FNB) and sciatic nerve block (SNB) improve postoperative pain management [1,8,9], if a drop foot occurs, it can be clinically challenging for orthopedic surgeons to distinguish whether it is associated with the preoperative SNB or various surgical factors. The incidence of common peroneal nerve palsy after TKA is 0.4% [10]. Drop foot resulting from the SNB resolves spontaneously as local anesthetics metabolize. Conversely, symptoms associated with the surgical procedure may persist longer, necessitating early intervention. However, solely administering a single FNB proved insufficient for postoperative pain relief when compared to the combination of FNB with SNB [11-13].

Therefore, we conducted postoperative SNB after verifying the patient’s ability to dorsiflex the ankle joint and ensuring the absence of motor impairments attributable to the surgery. Moreover, the subgluteal approach for SNB is standard practice to avoid the surgical wound. However, an ultrasound-guided nerve block at this level can be technically difficult because the sciatic nerve travels in the deep plane.

Karmarker et al.’s findings highlighted the simplicity of the subgluteal approach for SNB, involving the injection of local anesthetic beneath the gluteus major muscle at the plane of the greater trochanter and ischial tuberosity, in comparison to established methods [14]. In our hospital, the SNB at the subgluteal space was administered, and the effects of this more accessible approach for patients who underwent TKA were evaluated.

## Materials and methods

This study was approved by the Teikyo University Ethical Review Board for Medical and Health Research Involving Human Subjects (approval number: 21-073). The informed consent form for this study was published on our hospital website. Patients who underwent TKA under general anesthesia between May 2018 and June 2019 at the Teikyo University School of Medicine were screened for inclusion. Electronic medical and anesthesia records were used as the primary data sources. Patients undergoing TKA received intra-articular local anesthesia comprising 20 mL of 0.75% ropivacaine, 40 mg of methylprednisolone, and 0.05-0.5 mL of adrenaline during the surgical procedure. Intravenous patient-controlled analgesia (iv-PCA) of fentanyl was injected at a rate of 7.5 to 22.5 μg/hour, depending on the patient’s body weight. The exclusion criteria were as follows: emergency surgery, reoperation, received epidural anesthesia, did not receive intra-articular local anesthesia during surgery, did not receive a nerve block, did not receive iv-PCA, and underwent SNB at an area other than the subgluteal space.

The enrolled patients were divided into two groups, namely, the FNB alone group (control group), comprising those who underwent preoperative FNB without receiving SNB, and the post-SNB group, comprising individuals who underwent both preoperative FNB and postoperative SNB using the subgluteal space approach. All FNB procedures were performed under general anesthesia. Postoperative SNB was performed after recovery from anesthesia and after confirming the absence of postoperative motor impairment caused by surgery. Ropivacaine (10-40 mL, 0.2%-0.5%) was used for the nerve blocks.

Patient age, sex, body mass index, anesthesia method (total intravenous anesthesia or inhalation anesthesia), operative time, anesthesia time, intraoperative fluid volume, intraoperative blood loss, type of nerve block performed, use of local anesthetic, intraoperative opioid dose, and intraoperative antiemetic dose were extracted. The primary outcome was the pain-related Numerical Rating Scale (NRS) scores at the time of hospitalization, postoperatively in the recovery room, at the time of return to the hospital ward, and at 6, 12, 18, and 24 hours after surgery. The secondary outcomes included the postoperative use of antiemetics, iv-PCA suspension, and PONV and postoperative complications. PONV, postoperative use of antiemetics, and iv-PCA suspension data were extracted until the end of iv-PCA use. Postoperative complications included in-hospital mortality, irreversible neurological complications, local anesthetic intoxication, and reoperation after TKA. All patients received treatment following the clinical pathway for TKA, which routinely involved the administration of acetaminophen (1,800 mg/day) and/or non-steroidal anti-inflammatory drugs (NSAIDs). NSAIDs included celecoxib (400 mg/day) and flurbiprofen axetil (50 mg/day). Doses were adjusted or omitted according to patients’ hepatic and renal function. If patients did require pain medication, these routine drugs were passed. For those requesting additional pain relief, acetaminophen (1,000 mg/once) or NSAIDs (flurbiprofen axetil 50 mg/once) were administered, taking into account hepatic and renal function. The frequency of acetaminophen and NSAID usage was tallied from the end of the surgery to the first postoperative day. The NRS scores were recorded by a nurse during routine visits. There was no record of the number of times PCA was used by a patient because we used a disposable iv-PCA instrument that did not record the number of times PCA was used. The remaining drug volume of iv-PCA was checked when a nurse visited the hospital ward. Patients with PONV were treated with 10 mg of metoclopramide, and their iv-PCA was discontinued at the physician’s discretion if any complications occurred or if their PONV was not improved by medicine.

Continuous outcomes were compared using Student’s t-test, and categorical data were compared using the chi-square test. A multivariate analysis was performed using the logistic regression model method. Statistical significance was set at p < 0.05.

## Results

**Figure 1:**
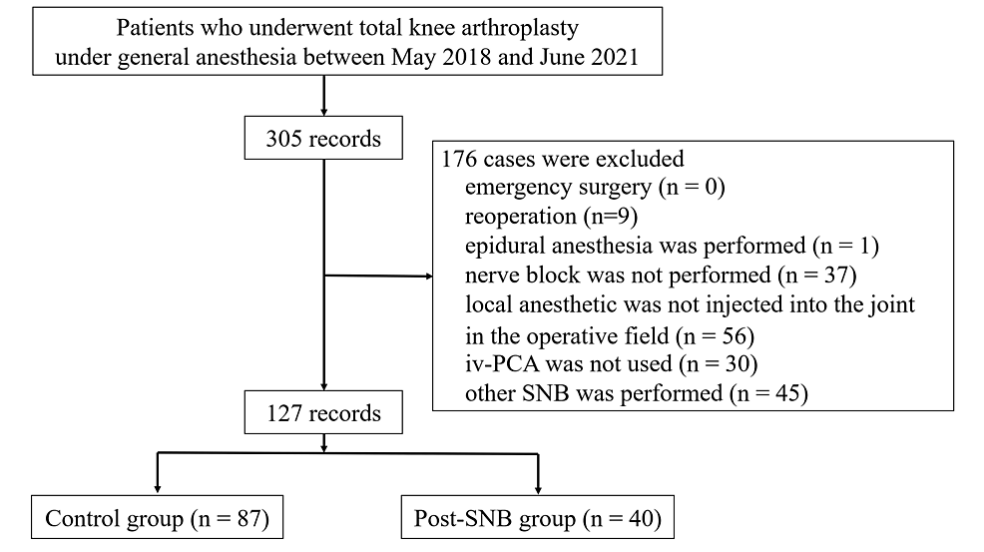
Flowchart of screened, excluded, and analyzed data. The control group included patients who received a preoperative femoral nerve block (FNB) but did not receive a sciatic nerve block (SNB). The post-SNB group included patients who received a preoperative FNB and a postoperative SNB using the subgluteal space approach.

The medical records of 305 patients who underwent TKA during the study period were screened. Emergency surgery was not performed. Reoperation was performed for nine cases, and epidural anesthesia was used for one case. The nerve block was not performed for 37 cases. Local anesthetic was not injected into the joint in the operative field for 56 cases, and iv-PCA was not used for 30 cases. Forty-five patients underwent SNB via the subgluteal or popliteal approach, selective tibial nerve block, or infiltration between the popliteal artery and the capsule of the knee block. Finally, of these 305 patients, 127 were included in this study based on the inclusion and exclusion criteria (87 patients in the control group and 40 patients in the post-SNB group) (Figure 1).

Baseline characteristics

To prevent PONV, we administered dexamethasone (0-6.6 mg) during the operation and included droperidol (0-5 mg) into the iv-PCA. No significant differences were observed in patient age, sex, body mass index, anesthesia method (inhalation anesthesia or total intravenous anesthesia), intraoperative dexamethasone doses, intraoperative doses of fentanyl, iv-PCA fentanyl doses, iv-PCA droperidol doses, duration of iv-PCA use, postoperative use of analgesics (acetaminophen and NSAIDs), time to first drug use for pain, operative time, anesthesia time, intraoperative infusion volume, or intraoperative blood loss between the groups (Table 1). The dose and concentration of the local anesthetic used for FNB were significantly higher in the control group compared to the post-SNB group. Additionally, the total amount of local anesthetic administered was significantly greater in the post-SNB group than in the control group (Table 1). The routine use of acetaminophen and NSAIDs did not exhibit significant differences between both groups. Similarly, the number of acetaminophen and/or NSAIDs for pain relief and the duration of first drug use for pain relief did not exhibit significant differences (Table 2).

**Table 1:** Baseline characteristics. Post-SNB group: patients who received a preoperative FNB and a postoperative SNB using the subgluteal space approach. Control group: patients who received a preoperative FNB but did not receive an SNB. Continuous outcomes were compared using Student’s t-test, and categorical data were compared using the chi-square test. FNB: femoral nerve block; SNB: sciatic nerve block; iv-PCA: intravenous patient-controlled analgesia

	Post-SNB group (n = 40)	Control group (n = 87)	P-value
Age, year, mean (SD)	74.30 (9.30)	75.07 (8.69)	0.651
Female, n (%)	30 (75.0)	68 (78.2)	0.868
Body mass index, kg/m^2^, mean (SD)	25.68 (4.36)	26.17 (4.02)	0.549
Inhalation anesthesia, n (%)	32 (80.0)	79 (90.8)	0.09
Total intravenous anesthesia, n (%)	8 (20.0)	8 (9.2)	0.09
Intraoperative doses of dexamethasone, mg, mean (SD)	3.30 (2.69)	2.59 (2.63)	0.162
Intraoperative doses of fentanyl, μg, mean (SD)	261.88 (97.56)	278.74 (116.11)	0.427
Fentanyl doses of iv-PCA, μg/hour, mean (SD)	15.00 (0.00)	15.14 (1.49)	0.545
Droperidol doses of iv-PCA, mg, mean (SD)	1.50 (1.58)	1.79 (1.93)	0.408
Duration of using iv-PCA, hour, mean (SD)	22.16 (2.62)	21.49 (5.25)	0.462
Operative time, minute, mean (SD)	127.70 (22.16)	129.49 (20.46)	0.656
Anesthetic time, minute, mean (SD)	189.59 (24.03)	182.95 (26.68)	0.233
Infusion volume, mL, mean (SD)	948.90 (266.68)	895.91 (235.04)	0.351
Blood loss, mL, mean (SD)	59.44 (53.06)	84.70 (91.16)	0.151
Local anesthetic concentration of FNB, %, mean (SD)	0.30 (0.03)	0.33 (0.07)	0.008
Local anesthetic dose of FNB, mg, mean (SD)	57.25 (9.23)	71.79 (23.55)	<0.001
Total local anesthetic dose, mg, mean (SD)	134.37 (21.3)	71.79 (23.55)	<0.001

**Table 2:** Acetaminophen and NSAID uses of the groups. NSAIDs include celecoxib or lopion. NRS: Numerical Rating Scale; NSAID: non-steroidal anti-inflammatory drug; SNB: sciatic nerve block

	Post-SNB group (n = 40)	Control group (n = 87)	P-value
Acetaminophen, mg, mean (SD)	600 (0.00)	600 (0.00)	NaN
Number of acetaminophen uses, n, mean (SD)	3.18 (0.58)	3.21 (0.47)	0.728
NSAIDs, mg, mean (SD)	200.00 (0.00)	193.53 (30.26)	0.211
Number of NSAID uses, n, mean (SD)	2.34 (0.48)	2.16 (0.44)	0.06
Acetaminophen for pain, mg, mean (SD)	1000.00 (0.00)	978.18 (80.06)	0.479
Number of acetaminophen uses for pain, n, mean (SD)	0.70 (0.88)	0.56 (0.69)	0.347
NSAIDs for pain, mg, mean (SD)	50.00 (0.00)	50.00 (0.00)	NaN
Number of NSAIDs uses for pain, n, mean (SD)	0.42 (0.78)	0.53 (0.78)	0.486
Number of drug uses for pain, n, mean (SD)	1.12 (1.32)	1.09 (1.18)	0.888
Time to first drug use for pain, hour, mean (SD)	15.60 (11.50)	12.63 (11.72)	0.31

Outcomes

The NRS score, which was the primary outcome of this study, did not differ significantly at any stage between groups (Table 3). Twenty-eight patients in the control group and five patients in the post-SNB group experienced PONV (Table 4); this difference between groups was significant (chi-square test; p = 0.03). The variables used for the logistic regression analysis of PONV between the post-SNB and control groups in block type were age, sex, anesthesia method, intraoperative dexamethasone doses, intraoperative fentanyl doses, iv-PCA fentanyl doses, iv-PCA droperidol doses, and duration of iv-PCA use. The analysis revealed a significant difference in iv-PCA droperidol doses and block type (Table 5). Seven patients in the control group and one patient in the post-SNB group discontinued iv-PCA because of PONV. No significant complications occurred in either group.

**Table 3:** NRS scores of the groups. NRS 1: score at admission; NRS 2: score in the postoperative recovery area; NRS 3: score at the time of return to the hospital ward; NRS 4: score six hours after surgery; NRS 5: score 12 hours after surgery; NRS 6: score 18 hours after surgery; NRS 7: score 24 hours after surgery. The chi-square test was used for categorical data. NRS: Numerical Rating Scale; SNB: sciatic nerve block

NRS	Post-SNB group (n = 40)	Control group (n = 87)	P-value
NRS 1 (mean)	4.10 (1.72)	4.42 (2.11)	0.406
NRS 2 (mean)	6.33 (2.38)	6.63 (3.01)	0.738
NRS 3 (mean)	3.24 (2.79)	4.12 (2.42)	0.083
NRS 4 (mean)	2.61 (1.91)	2.70 (2.07)	0.850
NRS 5 (mean)	3.30 (1.77)	2.39 (2.13)	0.246
NRS 6 (mean)	2.91 (2.27)	3.09 (2.02)	0.677
NRS 7 (mean)	3.24 (2.02)	3.41 (2.31)	0.725

**Table 4:** Data regarding PONV and iv-PCA suspension. The chi-square test was used for categorical data. SNB: sciatic nerve block; iv-PCA: intravenous patient-controlled analgesia; PONV: postoperative nausea and vomiting

	Post-SNB group (n = 40)	Control group (n = 87)	P-value
PONV, n (%)	5 (12.5)	28 (32.2)	0.033
iv-PCA suspension, n (%)	1 ( 2.7)	7 ( 8.8)	0.417
Use metoclopramide for PONV, n (%)	4 (10.8)	17 (20.7)	0.292
Total fentanyl dose of used iv-PCA, μg, mean (SD)	332.42 (39.33)	322.97 (80.08)	0.498

**Table 5:** Multivariate analysis of risk factors for PONV. The variables included were age, sex, anesthesia type (inhalation anesthesia or total intravenous anesthesia), intraoperative fentanyl dose, intraoperative dexamethasone dose, fentanyl doses of iv-PCA, droperidol doses of iv-PCA, and block type. PONV: postoperative nausea and vomiting; iv-PCA: intravenous patient-controlled analgesia

Variables used for the logistic regression	Odds ratio	95% confidence interval	P-value
Age	1.02	(0.95, 1.09)	0.54
Sex	3.75	(0.86, 1.63)	0.08
Anesthesia	0.82	(0.15, 4.50)	0.82
Intraoperative fentanyl doses	1	(0.99, 1.00)	0.31
Intraoperative dexamethasone doses	0.96	(0.77, 1.20)	0.71
Fentanyl doses of iv-PCA	1.2	(3.93e-132, 3.65e+129)	0.99
Droperidol doses of iv-PCA	0.64	(0.46, 0.89)	0.008
Duration of using iv-PCA	0.92	(0.83, 1.03)	0.15
Block type	0.47	(0.25, 0.88)	0.02

## Discussion

Despite our anticipation that analgesia in the sciatic nerve innervated region would enhance postoperative NRS scores, no significant differences were observed between the groups during this study. This result was inconsistent with that of a previous study suggesting that single-shot SNB reduces postoperative pain [8,11-13]. When using this approach, the fascial plane between the gluteus maximus and quadratus femoris should be identified with ultrasound, and the sciatic nerve itself is not always examined. This makes this block easier than the traditional SNB subgluteal approach, in which the sciatic nerve should be visualized, and a nerve stimulator is used to increase the success rate. To cover the sciatic nerve located in the subgluteal space, we used a relatively large local anesthetic volume (mean volume was 25.8 mL). However, there is a potential risk of incorrect injections, such as those directed into the gluteus maximus or at a distance from the sciatic nerve. This is due to the absence of clear visualization of the sciatic nerve using an echo or the use of a nerve stimulator during the SNB procedures. Anatomically, despite the sciatic nerves typically passing beneath the gluteus maximus muscle, instances of abnormal sciatic nerve pathways and pelvic bifurcation have been reported. Approximately 15% of cases involve abnormal travel of the sciatic nerve, and in some instances, the nerve may divide in the pelvis [15]. When the sciatic nerve travels abnormally, the efficacy of the block may be compromised. Additionally, technical challenges faced by the anesthesiologist performing nerve blocks, such as insufficient visualization of the needle tip, could contribute to inadequate postoperative analgesia.

The study results revealed a significantly lower incidence of PONV in the post-SNB group compared to the control group. Notably, the use of dexamethasone and droperidol, known for their efficacy in preventing PONV [16,17], did not differ between the groups. Additionally, the risk factors for PONV, including age, sex, intraoperative fentanyl dose, and amount of fentanyl administered for iv-PCA, did not exhibit significant differences.

Logistic regression analysis confirmed that postoperative SNB remained independently associated with PONV occurrence, even after adding the risk factors of PONV. The causal relationship between SNB and PONV remains unclear based on prior studies [13,18,19]. Clinically, discontinuation of iv-PCA due to PONV can exacerbate postoperative pain. This simple SNB may contribute to multimodal analgesia by reducing PONV.

The control group utilized a higher local anesthetic dose for FNB compared to the post-SNB group. This difference may be attributed to the post-SNB group requiring local anesthesia for both preoperative FNB and postoperative SNB, whereas the control group only needed it for FNB. Consequently, anesthesiologists in the control group might have employed a higher concentration of local anesthetics to achieve a more dense nerve block.

This study involved three major limitations. First, there was a substantial difference in sample size between the control and post-SNB (87 and 40) groups. In a study of this scale, such a discrepancy could pose a significant concern that cannot be overlooked. Second, all patients in this study underwent FNB and received intra-articular local anesthesia during surgery, iv-PCA, and regular administration of acetaminophen and NSAIDs. It is possible that the effects of the SNB subgluteal space approach may have been masked by adequate pain control through this multimodal analgesia. Moreover, our iv-PCA utilized disposable instruments, preventing us from accurately counting the number of times PCA was used, necessitating additional analgesia. Consequently, this makes it difficult to detect the difference in analgesia between the groups. Finally, the method to evaluate the NRS was not standardized. For instance, pain assessment was not differentiated between the femoral and sciatic nerve areas, making it challenging to determine the effectiveness of FNB. This limitation suggests that insufficient FNB in the post-SNB group could have reduced the effect of SNB, leading to a non-significant difference in NRS between the two groups. The presence of missing data resulting from these limitations may compromise the robustness of the study results.

## Conclusions

The preoperative FNB and postoperative SNB using the subgluteal space approach did not result in a significant analgesic effect for patients who underwent TKA. However, it may reduce PONV compared to the preoperative FNB alone.
